# Moringin from *Moringa Oleifera* Seeds Inhibits Growth, Arrests Cell-Cycle, and Induces Apoptosis of SH-SY5Y Human Neuroblastoma Cells through the Modulation of NF-κB and Apoptotic Related Factors

**DOI:** 10.3390/ijms20081930

**Published:** 2019-04-19

**Authors:** Santa Cirmi, Nadia Ferlazzo, Agnese Gugliandolo, Laura Musumeci, Emanuela Mazzon, Alessia Bramanti, Michele Navarra

**Affiliations:** 1 Department of Chemical, Biological, Pharmaceutical and Environmental Sciences, University of Messina, 98168 Messina, Italy; scirmi@unime.it (S.C.); nferlazzo@unime.it (N.F.); lauramusumeci93@gmail.com (L.M.); 2 IRCCS Centro Neurolesi “Bonino-Pulejo”, 98124 Messina, Italy; agnese.gugliandolo@irccsme.it (A.G.); emanuela.mazzon@irccsme.it (E.M.); alessia.bramanti@gmail.com (A.B.); 3 Eduardo Caianiello Institute of Applied Science and Intelligent Systems (ISASI), National Research Council, 98100 Messina, Italy

**Keywords:** moringin, glucosinolates, isothiocyanate, cancer, SH-SY5Y cells, *Moringa oleifera*, apoptosis

## Abstract

In the last decades, glucosinolates (GLs), precursors of isothiocyanates (ITCs), have been studied mostly for their chemopreventive and chemotherapeutic properties. The aim of our research was to study the antiproliferative effect of 4-(α-L-rhamnopyranosyloxy) benzyl glucosinolate (glucomoringin; GMG) bioactivated by myrosinase enzyme to form the corresponding isothiocyanate 4-(α-L-rhamnopyranosyloxy) benzyl C (moringin) in SH-SY5Y human neuroblastoma cells. We found that moringin significantly reduced SH-SY5Y cell growth in a time and concentration-dependent (*p* < 0.05, 0.01, and 0.001 vs. ctrl, after treatment with 16.4 µM moringin for 24, 48, and 72 h, respectively) manner through a mechanism involving the activation of apoptotic machinery. In addition, it altered the normal progression of cells through the cell cycle, increasing the cell population in both G2 and S phases, as well as decreasing that in the G1 phase. Studying the drug mechanism of action, we found that moringin was able to increase the expression of p53, p21, and Bax at both the protein and transcriptional level. Moreover, exposure of SH-SY5Y cells to moringin significantly increased the gene expression of both caspase 3 and 9 and enhanced their cleavage, thereby initiating an intrinsic apoptotic cascade. Finally, moringin inhibited nuclear translocation of NF-κB. Our study demonstrates the ability of moringin to reduce the growth of SH-SY5Y cells and reveals its mechanism of action, suggesting its promising role as an anticancer drug.

## 1. Introduction

*Moringa oleifera* Lam. is the most widely distributed plant of the Moringaceae family that grows widely in many tropical and subtropical countries [1]. Commonly called by the name of ‘the miracle tree’, it is a multi-use plant used as a functional food for human nutrition, animal feeding, and for medicinal purposes [2]. The majority of its medicinal and nutritional properties have been ascribed to some parts of the plant, such as seeds, flowers, roots, leaves and bark, which are used in traditional medicine for the management of several diseases [3]. Indeed, extracts of different parts of *Moringa oleifera* have been recognized as anti-inflammatory, anti-bacterial, anti-cancer, and hepatoprotective remedies [4,5]. Moreover, *M. oleifera* is a source of several micronutrients, phenolic compounds, and glucosinolates (GLs). Generally, GLs have three moieties: a β-thioglucose moiety, a sulfonated oxime moiety, and a variable aglycone side chain derived from an α-amino acid [6]. Furthermore, *M. oleifera* possesses many unusual GLs with atypical characteristics due to a second saccharide residue in the aglyconic side chain [6,7].

In the last decades, GLs precursor, isothiocyanates (ITCs), have been studied mostly due to their chemopreventive and chemotherapeutic properties [8]. Observational studies have shown that the consumption of GLs/ITCs-rich cruciferous vegetables protects against several types of human cancer by induction of both apoptosis and cell cycle arrest. These anticancer properties have been attributed to the high content of naturally occurring ITCs [9]. The principal GL in *Moringa oleifera* is the 4-(α-L-rhamnopyranosiloxy)benzyl glucosinolate, also called glucomoringin. Due to its unusual structure, this compound may have biological properties different from other GLs [7].

Neuroblastoma (NB) is the most common extra-cranial solid tumor of early childhood accounting for about 28% of all cancers diagnosed in infants in the US and Europe. Annually, about 700 cases occur in Canada and the USA as well as 1500 in Europe [10]. Even if aggressive and intensive care had some improvements in the cure rate of NB patients, their prognosis is still poor. Moreover, conventional cancer therapies cause serious side effects and, often, merely extend the patient’s lifespan by a few years. Therefore, natural products to prevent cancer, and alternative approaches to its treatment are escalating. For this purpose, due to the role of ITCs in cancer management, the aim of our study was to evaluate the antiproliferative effect of moringin on SH-SY5Y human neuroblastoma cells, and its molecular mechanisms of action. The natural drug resulted from myrosinase-catalyzed quantitative hydrolysis of glucomoringin purified from the seeds of the *Moringa oleifera*. 

## 2. Results

### 2.1. Moringin Inhibits the Growth of SH-SY5Y Human Neuroblastoma Cells

**Figure 1:**
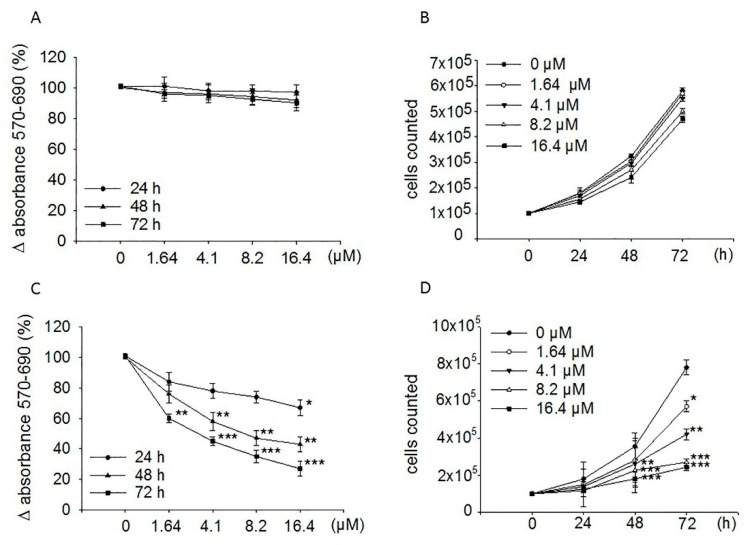
Effects of moringin on the proliferation of WI-38 and SH-SY5Y cells. Both WI-38 (**A**,**B**) and SH-SY5Y (**C**,**D**) cells were exposed to the drug (1.64–16.4 μM) for the indicated times. Proliferation rate was performed by the MTT assay (**A**,**C**) and cell count (**B**,**D**). MTT results are expressed as percentages ± SEM of absorbance detected in treated cells. Each concentration was eightfold tested, and three independent experiments were carried out. Data from the cell counts were expressed as mean ± SEM of three independent experiments performed in triplicate. *p < 0.05, **p < 0.01, and ***p < 0.001 vs. control, respectively.

Figure 1C shows that treatment of SH-SY5Y with moringin for 24, 48, and 72 h reduced cells proliferation in a concentration-dependent manner, achieving the greatest inhibitory effect (73%; *p* < 0.001) after 72 h of exposure to 16.4 µM concentration. However, it was already active at 48 (57%; *p* < 0.01) and 24 h (33%; *p* < 0.05) of incubation. Furthermore, it is active at a concentration of 1.64 µM. MTT data were established by counting cells in a Neubauer hemocytometer chamber after 24, 48, and 72 h treatment with moringin (Figure 1D). The IC_50_ value at 72 h of exposure was 1.7 µM. Contrariwise, ITC did not affect the proliferation of the WI-38 diploid fibroblast cell line (Figure 1A,B).

### 2.2. Cytotoxic Effect Induced by Moringin

**Figure 2:**
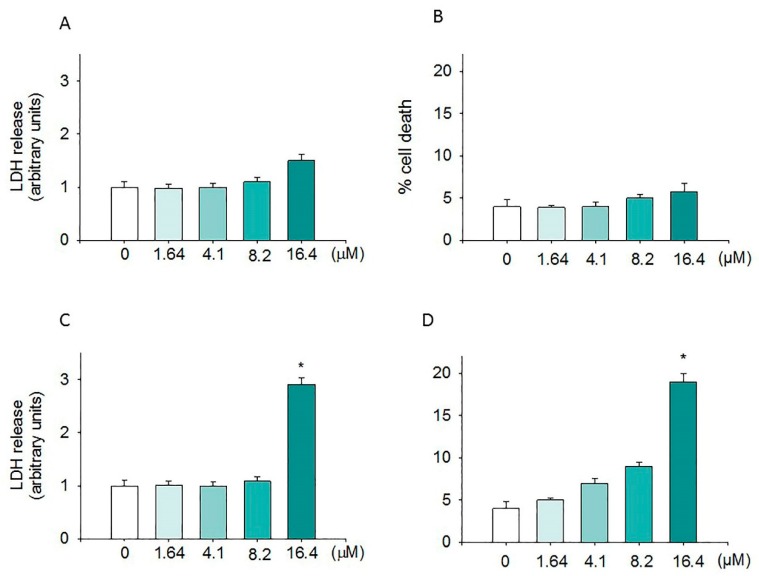
Cytotoxic effects of moringin. The cytotoxic activity of moringin (1.64–16.4 μM) was evaluated in terms of both LDH release (**A**,**C**) and cell death (**B**,**D**) after 24 h of exposure. LDH levels were extrapolated as the values detected in control cells which were arbitrarily expressed as 1. Cell death was reported as the percentage of blue stained (non-viable) vs. total cells counted. Data, expressed as mean ± SEM, represent the values obtained in three different sets of experiments made in triplicate. * *p* < 0.05 vs. control.

**Figure 3:**
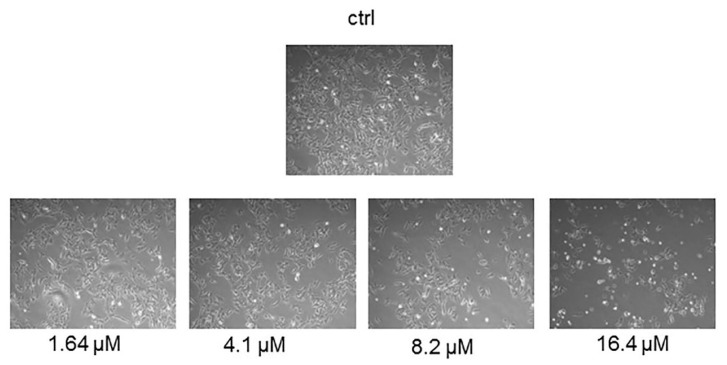
Morphological analyses of the SH-SY5Y cells treated with moringin. Cell morphology was monitored after treatment with different concentrations (1.64–16.4 μM) of moringin for 24 h. The morphological changes were observed under an inverted microscope (200×).

In order to evaluate if the anti-proliferative effect induced by moringin was due to a cytotoxic effect, the SH-SY5Y cells were exposed to different concentrations of the compound for 24 h, and then the LDH assay and trypan blue test were performed. Figure 2 shows that, at the concentrations ranging from 1.64 to 8.2 µM, moringin did not cause SH-SY5Y cell death (Figure 2C) or increase LDH release (Figure 2D). On the contrary, the highest concentration of moringin tested in this study (16.4 µM) induced significant cytotoxic effects (*p* < 0.05 and *p* < 0.01; Figure 2C,D) on SH-SY5Y cells. Figure 2A,B show that moringin did not induce a significant increase in WI-38 cell death. Moreover, as illustrated in Figure 3, exposure to increasing concentrations of moringin for 24 h altered the SH-SY5Y cell morphology, which acquired a rounded shape (index of cellular suffering), detaching themselves from the bottom of the well.

### 2.3. Moringin Induced Apoptosis in SH-SY5Y Neuroblastoma Cells

**Figure 4:**
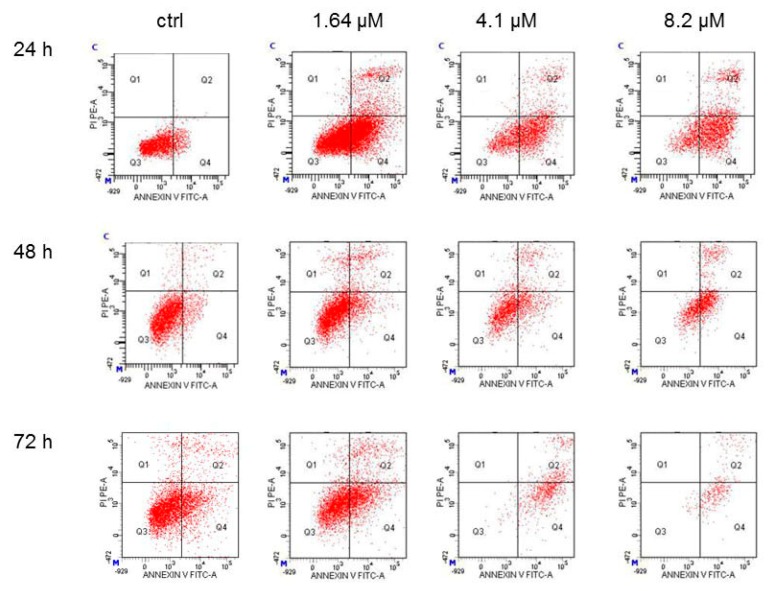
Cytofluorimetric evaluation of apoptosis on the SH-SY5Y cells exposed to moringin. Detection of apoptosis was performed by the Annexin V test. Representative Annexin V vs. PI dot plots of the SH-SY5Y cells treated with 1.64–8.2 μM of moringin for 24–72 h are shown. Q3 contains the viable cells, Q4 the cells in early apoptosis, Q2 the cells in late apoptosis, and Q1 contains the necrotic cells. The FACS analysis presented is representative of three different experiments.

To address the way by which moringin reduced the SH-SY5Y cell growth, apoptosis was detected using cytofluorimetric anlysis. After 24 h of treatment with 8.2 μM of moringin, apoptosis was found in 78% of the cell population, which further reached 90% after an elongated duration (72 h) of exposure to the cells (Figure 4). Moreover, the 16.4 μM concentration reduced the number of cells to such a level that did not allow a correct interpretation of the results (data not shown).

### 2.4. Moringin Induced the Accumulation of G2 Phase in SH-SY5Y

**Figure 5:**
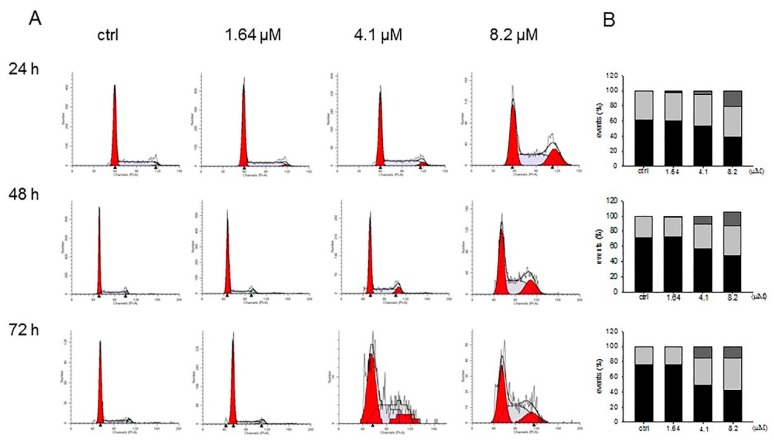
Influence of moringin on cell cycle distribution in SH-SY5Y cells. Progression of SH-SY5Y cells through the cell cycle was examined by flow cytometry analysis after exposure to moringin for 24–72 h (**A**). The histograms on the right (**B**) represent the percentage of events in G1 (black bar), G2 (grey), and S (light grey) phases and are the mean ± SEM of three independent experiments.

Since Brunelli et al. (2010) reported that moringin caused a strong cell cycle perturbation in the RPMI-8226 human myeloma cell line, we analyzed its effect in the SH-SY5Y cell cycle distribution. As shown in Figure 5, moringin ranging from 1.64 to 8.2 μM increased the cell population in both G2 and S phases, while decreased the number of cells in the G1 phase. The effects of ITC are evident already after 24 h of exposure and remained unchanged for the following 48 h.

### 2.5. Effects of Moringin on the Apoptotic Pathway

Apoptosis is tightly regulated by several factors, including tumor suppressors and inducer genes, such as the Bcl-2 family proteins, while the caspases (cysteine aspartyl protease) are considered the most important executors of programmed cell death. In order to investigate the mechanism through which moringin induces SH-SY5Y apoptotic cell death, we evaluated the expression of the main proteins involved in the regulation of apoptosis by both western blot analysis and Real-Time PCR.

**Figure 6:**
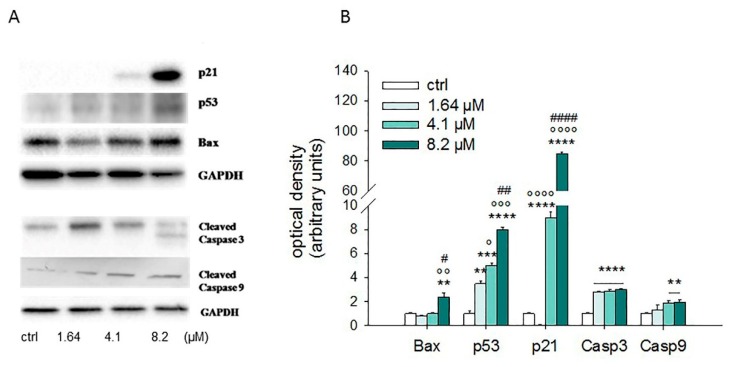
Effects of moringin on expression of apoptosis-related proteins. (**A**) Immunoblot of SH-SY5Y cells exposed to 1.64–8.2 μM of moringin for 24 h: results of a representative experiment are shown. (**B**) Densitometric analysis of immunoreactive bands from three independent blots are reported. Levels are extrapolated as values detected in control cells, which are arbitrarily assigned as 1. ** *p* < 0.01, *** *p* < 0.001, and **** *p* < 0.0001 vs. untreated culture; ° *p* < 0.05, °° *p* < 0.01, °°° *p* < 0.001, and °°°° *p* < 0.0001 vs. moringin 1.64 µM; ^#^ *p* < 0.05, ^##^ *p* < 0.01, and ^####^ *p* < 0.0001 vs. moringin 4.1 µM.

**Figure 7:**
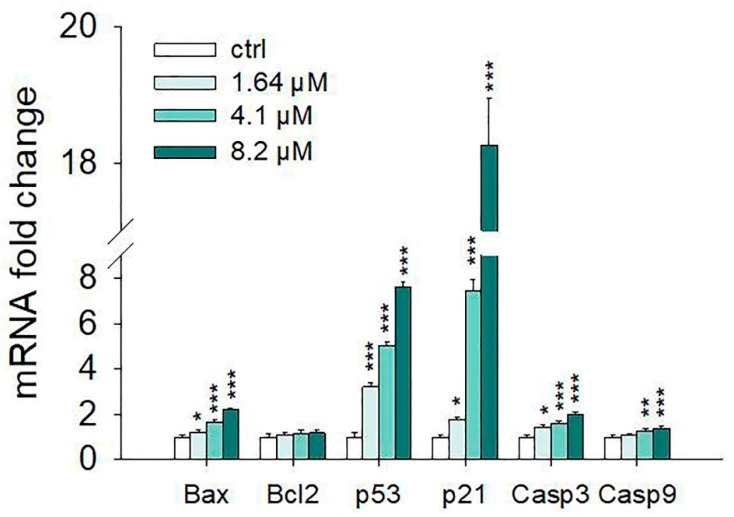
Effects of moringin on mRNA levels of apoptosis-related genes. SH-SY5Y cells were exposed to 1.64–8.2 μM of moringin for 24 h and then subjected to Real-Time PCR. Relative quantities of mRNA were calculated by using the 2^–ΔΔCT^ method. Results are expressed as fold change relative to untreated cells. Data, expressed as mean ± SEM, represent the values obtained in three different sets of experiments made in triplicate. * *p* < 0.05, ** *p* < 0.01, and *** *p* < 0.001 vs. control, respectively.

Western blot analyses performed after 24 h of exposure to 1.64–8.2 µM of moringin showed that it was able to increase the protein level of p53, p21, Bax, cleaved caspase 3, and 9 (Figure 6). The results of the Real-Time PCR experiments strengthen this data (Figure 7). In particular, moringin 8.2 µM showed a 1.5-fold increase in Bax at both gene (*p* < 0.001) and protein (*p* < 0.01) levels, while mRNA of Bcl-2 did not change significantly. Moreover, moringin 8.2 µM significantly raised the expression of the gene encoding protein p53 (*p* < 0.001), as well as increasing its protein amount (*p* < 0.0001). However, p21 was the apoptotic target most affected by moringin, with a dramatic increase at both the protein and transcriptional level, especially when treated with 4.1 and 8.2 µM. Its gene expression was 7 and 18 times higher than the control when the SH-SY5Y cells were treated with moringin at 4.1 and 8.2 µM concentrations (*p* < 0.001), respectively, as well as the protein levels which were increased by 9- and 85-fold when subjected to the same treatments compared to the control (*p* < 0.0001). Finally, we investigated the role of caspases in the apoptotic cell death induced by moringin. As shown in Figure 7, exposure of SH-SY5Y cells to moringin significantly increased the gene expression of both caspase 3 (4.1 µM and 8.2 µM, *p* < 0.001) and 9 (4.1 µM *p* < 0.01, 8.2 µM *p* < 0.001) and enhanced their cleavage, thereby initiating the intrinsic apoptotic cascade.

### 2.6. Effect of Moringin on NF-κB

**Figure 8:**
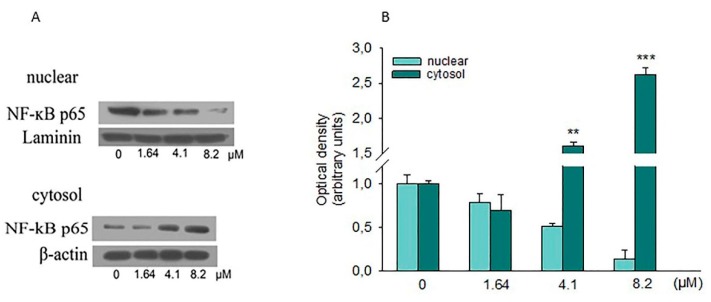
Effects of moringin on NF-ĸB activation. (**A**) SH-SY5Y cells were treated for 24 h with the indicated concentration of moringin, and then, cytoplasmic and nuclear proteins were analyzed by Western blot for the NF-ĸB p65 sub-unit. (**B**) Densitometric analysis of autoradiographic bands in which levels of the nuclear protein were normalized for laminin and the cytosolic ones for β-actin. A representative immunoblot of three independent experiments is shown. * *p* < 0.01, and *** *p* < 0.001 vs. control, respectively.

NF-κB, associated with IκB, is found in the cytoplasm as an inactive complex. When some stimuli occur, these can induce the dissociation of the NF-κB/IκB complex through the degradation of the IκB subunit, thus permitting the translocation of NF-κB in the nucleus. There, it binds to specific sequences of DNA, regulating the transcription of several target genes. Substantial evidence indicates that NF-κB is involved in different molecular pathways associated with tumorigenesis. Therefore, we investigated if NF-κB has been involved in the inhibition of SH-SY5Y cell growth caused by moringin. Western blot analysis showed a reduction of the nuclear levels of p65 revealing the ability of moringin to inhibit its nuclear translocation (Figure 8).

## 3. Discussion

Numerous studies have demonstrated that natural products possess several biological activities widely used in traditional, folk, as well as complementary and alternative medicine [11,12,13]. Hence, many products from the plant kingdom have been widely investigated for their therapeutic effects, and some of these have obtained clinical approval or are actually in clinical trials [14,15]. These include natural or semisynthetic drugs used in the field of cancer such as vinblastine, vincristine, topotecan, and irinotecan (camptothecin derivatives), etoposide (epipodophyllotoxin derivative) and paclitaxel, while others are under preclinical experimentation [16,17]. In this context, several phytochemicals, such as GLs and their breakdown products (i.e. ITCs), have been shown to prevent the risk of carcinogenesis [18,19]. ITCs are a group of natural products, which are not produced as such by the plant, but rather released after cell damage by the enzymatic action of myrosinase on their GLs precursors. ITCs have long been known for their anti-tumoral and anti-inflammatory activities. A consistent number of *in vitro* and *in vivo* studies have proved the preventive and therapeutic effects of various ITCs such as benzyl ITC, allyl ITC, phenethyl ITC, and sulforaphane against several types of cancers. They act by blocking the characteristic features of cancer including cell proliferation, invasion, angiogenesis, and metastasis as well as lead to cell cycle arrest and apoptosis [20].

Previously, *in vitro* and *in vivo* studies have shown the anticancer effect of moringin against different models of myeloma, carcinoma, astrocytoma, and leukemia [7,21]. Here, for the first time, we investigated the anti-proliferative effect of moringin in an *in vitro* model of neuroblastoma, showing that moringin reduced the growth of SH-SY5Y cells through different intracellular pathways leading to apoptotic cell death. Of note, up to 16.4 µM, moringin did not induce any cytotoxic effect, since it did not cause cell death or increase LDH release. Therefore, except for the highest concentration tested in this study, the antiproliferative effect of moringin in SH-SY5Y cells was not mediated by necrotic cell death, as was confirmed by the results of cytofluorimetric analyses. Interestingly, a previous study by Rajan and co-workers [21] showed that moringin did not affect the normal human periodontal ligament tissue-derived mesenchymal stem cell (hPDLSCs) viability, as well as those of the WI-38 fibroblasts employed in this study. However, 24 h exposure to moringin altered SH-SY5Y cell morphology, causing their round shape and detachment from the bottom of the well. A similar effect on cellular morphology was reported in human pancreatic cancer cells exposed to benzyl ITC [22].

Many cancer cells are unable to undergo apoptosis, and carcinogenesis increases when apoptosis is missing [23]. On the other hand, the goal of apoptosis is to avoid the proliferation of malignantly transformed cells that play a crucial role in cancer. Therefore, apoptosis is considered an important objective of several anticancer drugs. Data from the cytofluorimetric analysis performed in this study suggest that moringin arrested the cell cycle and induced apoptosis, suggesting the mechanism through which it exerts its antiproliferative activity.

It is well-known that p53 acts as a tumor suppressor, leading to G2 cell cycle arrest following DNA damage [24]. Multiple downstream-targeted genes, such as p21 and Bax, are upregulated by p53 [25]. p21 acts as a cyclin-dependent kinase inhibitory (CDKI) protein, with an affinity for both G1 and G2 cyclin-CDK complexes, highlighting the main mechanism for p53-mediated cell cycle arrest in G1 or G2 phases. However, in contrast to what was observed by Brunelli and co-workers [7] in A2780, NSCLC, H460 WT, and H460 S5 cells, we demonstrated that, in SH-SY5Y cells, moringin induced cell cycle arrest in the G2 phase, probably through the involvement of p53 and p21, as suggested by their upregulation which was induced by the moringin treatment at both the gene and protein levels. However, our finding is in line with those of other studies showing that another ITC, the allyl isothiocyanate, caused G2/M phase arrest in human brain malignant glioma GBM 8401 cells [26] and prostate cancer cells [27].

The apoptotic machinery is strictly controlled by numerous factors comprising tumor inducers and suppressor genes such as the Bcl-2 family proteins which could stimulate survival of tumor cells, thus conferring resistance to chemotherapy (Bcl-2 and Bcl-XL) or inducing apoptosis (Bax and Bad) [28]. Results from the Western blot analysis showed that treatment of SH-SY5Y cells with 4.1 and 8.2 µM concentrations of moringin for 24 h up-regulated some pro-apoptotic proteins such as Bax, p21, and p53. Moreover, the Real-Time PCR data demonstrated that moringin acts at transcriptional levels modifying the mRNA of genes related to the apoptotic process at 1.64 µM concentration. In addition, exposure of SH-SY5Y cells to moringin significantly increased the gene expression of both caspase 3 and 9 and enhanced their cleavage, thereby initiating the intrinsic apoptotic cascade.

The NF-κB family of transcription factors regulates growth, differentiation, and apoptosis in several tissues [29]. In its inactive form, NF-κB is present in the cytosol as an inducible multi-subunit complex composed of two protein subunits, p50 and p65 polypeptides that are complexed to a third NF-κB inhibitory subunit, IκB. The activation of NF-κB is frequent in several human malignancies, driving up-regulation of genes that codify for growth factors, anti-apoptotic genes, pro-inflammatory cytokines, and adhesion molecules [30,31]. Many studies have shown that the downregulation of NF-κB in the nucleus is associated with apoptosis. Hence, inhibition of NF-κB activation has been postulated as a key target for cancer chemoprevention [32,33]. Several natural products, such as ITCs, exert their anti-cancer effects through the suppression of one or more steps in the NF-κB signaling pathway. In this line, our data demonstrated that moringin inhibited nuclear translocation of NF-κB in SH-SY5Y cells, suggesting a key role of this transcription factor in the anti-adhesive and pro-apoptotic effect of moringin in SH-SY5Y cells. Our data are in contrast with what was reported by Giacoppo and co-workers [34] but in accordance with what was shown by Brunelli and collaborators [7] in the RAW-NFκB cell. Therefore, our results suggest that the antiproliferative effect of moringin observed in SH-SY5Y cells could be due, at least in part, to its suppressive effect on the NF-κB pathways and induction of cytoprotective genes.

Overall, our study demonstrates the ability of moringin in reducing the growth of SH-SY5Y cells, implying the knowledge on its mechanism of action, and suggests its promising role as an anticancer drug.

## 4. Materials and Methods

### 4.1. Sample Preparation

Glucomoringin (GMG) was isolated from *M. oleifera* L. (seed cake powder PKM2 provided by Indena India Pvt. Ltd.; Bangalore, India) at the CREA-AA laboratory in Bologna in two sequential steps, by anion exchange and size exclusion chromatography, according to a method previously described [35]. The purity was assayed by HPLC analysis of the desulfo-derivative, yielding GMG with a purity of about 99% (based on peak area value), and more than 95% on a weight basis, due to its high hygroscopic properties [35]. The enzyme Myrosinase (Myr) was extracted from seeds of the *Sinapis alba* L. as described by Pessina et al. [36] with some modification.

GMG powder was dissolved in culture media and then bioactivated with Myr (30 µL/mL of GMG solution) for 30 min at 37 °C to obtain the bioactive moringin (GMG-ITC). The total conversion of GMG into moringin was confirmed by HPLC analysis of the desulfo-derivative [37].

### 4.2. Cell Culture and Drug Treatment

Experiments were carried out using the tumor derived SH-SY5Y human neuroblastoma cell line as well as the fibroblast WI-38, derived from normal embryonic lung tissue. Both cell lines were originally obtained from ATCC (Rockville, MD, USA). The cells were grown in a monolayer at 37 °C in 5% CO2 humidified atmosphere. The SH-SY5Y cells were cultured in RPMI supplemented with 10% (*v*/*v*) heat-inactivated fetal bovine serum, L-glutamine (2 mM), sodium pyruvate (1 mM), penicillin (100 lU/mL), and streptomycin (100 μg/mL). WI-38 cell lines were grown in DMEM supplemented with 10% FBS, glutamine, and penicillin-streptomycin. All reagents were from Gibco (Life Technologies, Monza, Italy).

Experiments performed in this study were carried out by seeding the cells in the appropriate culture plates 24 h before each experiment. In particular, SH-SY5Y and WI-38 cells were plated in 96-well plates for both the 4,5-dimethylthiazol-2-yl)-2,5-diphenyltetrazolium bromide (MTT) test and the lactate dehydrogenase (LDH) assay (5 × 10^3^ and 15 × 10^3^ cell/well, respectively). The cell count assay, the trypan blue dye test, and the cytofluorimetric analyses were carried out on SH-SY5Y seeded onto a 6-well plate at a density of 10 × 10^3^ cells/well, while for the protein expression studies we plated 6 × 10^5^ cells/well. Finally, the mRNA profile was evaluated by seeding the SH-SY5Y in 100 mm Petri dishes at a density of 15 × 10^5^ cells/plate. The next day, growth media was replaced with fresh medium with or without (untreated cultures) increased concentrations of moringin ranging from 1.64 to 16.40 μM. The length of the treatment depended on the assay performed.

### 4.3. Cell Proliferation Assays

In order to evaluate the anti-proliferative activity of moringin, we performed both the MTT test and cell count assay as previously described [38]. SH-SY5Y and WI-38 cells were seeded and treated as described above. After 24, 48, and 72 h of incubation, the plates were centrifuged at 1200 rpm for 10 min, the supernatants were removed and fresh media without phenol red containing 0.5 mg/mL of MTT (Sigma-Aldrich, Milan, Italy), was added to each well. The plates were replaced in the incubator for 4 h and gently shaken occasionally. Then, the plates were centrifuged at 1200 rpm for 10 min, the supernatants were removed and crystals of formazan (MTT metabolic product) were solubilized in 100 µL of HCl/isopropanol 0.1 N lysis buffer. The absorbance was spectrophotometrically quantified by a microplate spectrophotometer (iMark™ microplate reader, Bio-Rad Laboratories, Milan, Italy) at a wavelength of 570 nm with reference at 690 nm. Differences in cell proliferation were measured as a percentage of growth rates of treated cells compared to untreated cultures.

Cell growth was also detected by the cell count assay. Briefly, SH-SY5Y and WI-38 cells were seeded and treated as described above and then harvested by trypsinization, centrifuged, and re-suspended in a known amount of culture medium. Aliquots of cell suspensions were put in a Neubauer hemocytometer chamber and the cells were counted by an optical microscope.

The proliferation assays were performed in eightplicate (MTT test) or triplicate (cell count) and repeated three different times.

### 4.4. Cytotoxicity Assays

Possible drug cytotoxicity was assessed by both the lactate dehydrogenase (LDH) assay and the trypan blue test after 24 h of treatment with moringin. LDH concentrations in the medium of treated and untreated cells were measured by a commercial kit (CytoTox 96^®^ Non-Radioactive Cytotoxicity Assay, Promega, Milan, Italy). Briefly, plates were centrifuged at 400× *g* for five minutes and then 50 µL of supernatant from each well was transferred to the corresponding wells of clean plates together with 50 µL of fresh prepared LDH reaction solution. The plates were put on an orbital shaker for 30 min at room temperature and then 50 µL of stop solution was added to each well. The absorbance was quantified spectrophotometrically at 490 nm. LDH levels were extrapolated as the values detected in control cells, which were arbitrarily expressed as 1 [39]. The trypan blue dye (0.4% *w*/*v*; TB) exclusion assay was used to detect dead cells, that were reported as the percentage of stained (non-viable) vs. total cells counted [39]. Both LDH and TB experiments were carried out in triplicate and repeated three times.

### 4.5. Cytofluorimetric Evaluation of Apoptosis

Cell death induced by moringin was also assessed by fluorescence-activated cell sorting (FACS) analysis exploiting the annexin-V/propidium iodide (PI) staining, a method that allows the distinction of early apoptosis from late apoptosis and necrosis. The PI is a DNA intercalator that stains necrotic cells because it binds to the DNA of cells with a damaged membrane. Indeed, PI cannot enters viable cells that keep membrane integrity. Annexin-V has a high affinity to phosphatidylserines (PS), a phospholipid component exposed on the outer leaflet of the plasma membrane of apoptotic cells. Our experiments were performed using a commercial kit (BD Biosciences, Milan, Italy) in which the annexin-V conjugated to fluorescein isothiocyanate (FITC) which serves as a sensitive probe of cells that are undergoing apoptosis, compared to PI which allows the discrimination of the early from late apoptotic cells. Therefore, based on the staining intensity, viable cells are both annexin-V and PI negative, early apoptotic cells are annexin-V positive and PI negative, late apoptotic cells are both annexin-V and PI positive, and necrotic cells are annexin-V negative and PI positive.

After 24, 48, or 72 h of treatment with moringin, the SH-SY5Y cells were collected by trypsinization, washed, centrifuged, and re-suspended in the binding buffer provided by the kit at 1 × 10^6^ cell/mL concentration. Then, both 5 µL of annexin-V-FITC and 10 µL of PI were added to 200 µL of each sample, gently vortexed, and incubated at room temperature in the darkness for 15 min. Finally, the samples were run on a Novocyte 2000 (ACEA Bioscences Inc., San Diego, California, USA) cytofluorimeter [40]. The experiment was repeated three times in triplicate.

### 4.6. Cell Cycle Analysis

Cytofluorimetric studies were also used to check the progression of cells through the cell cycle. The SH-SY5Y cells were treated for 24, 48, and 72 h, harvested and centrifuged for 10 min at 1200 rpm. Then, the cells were fixed in cold 70% ethanol at 4 °C for 2 h, washed with cold PBS, centrifuged, and re-suspended in 250 µL of PBS together with 5 µL of 10 mg/mL RNase A. After 1 h of incubation at 37 °C, 10 µL of 1 mg/mL of PI was added to each sample and the cell suspensions were run on flow cytometry [41]. The experiment was repeated three times in triplicate.

### 4.7. Western Blot Analysis

SH-SY5Y cells were seeded and treated for 24 h as described above, and then processed following Ferlazzo and co-workers [40]. Briefly, the cells were washed with ice-cold PBS and lysed in a buffer containing: 0.32 M sucrose, 10 mM Tris-HCl pH 7.4, 1 mM EGTA, 2 mM EDTA, 5 mM NaN3, 10 mM 2-mercaptoethanol, 50 mM NaF, and protease inhibitor (Roche Diagnostics Corporation, Indianapolis, USA). The homogenates were chilled on ice for 20 min, centrifuged at 9600× *g* at 4 °C for 1 min, and then the supernatant (cytosolic extract) was collected. Following, pellets were suspended in a lysis buffer containing 0.1% Triton X-100, 150 mM NaCl, 10 mM Tris-HCl, pH 7.4, 1 mM EGTA, 1 mM EDTA, and protease inhibitors (Roche Diagnostics Corporation), kept on ice for 30 min and centrifuged at 9600× *g* at 4 °C for 10 min. The supernatant (nuclear extract) was collected and stored at −80 °C until use. Protein concentrations were determined using a Bio-Rad Protein Assay (Bio-Rad Laboratories) using BSA as the standard. Proteins were separated on sodium dodecyl sulfate-polyacrylamide gel electrophoresis (SDS-PAGE) and transferred onto a PVDF transfer membrane (Immobilon-P PVDF, Merck Millipore division of Merck KGaA, Darmstadt, Germany), blocked with PBS containing 5% non-fat dried milk for 1 h at room temperature, and subsequently probed with the following antibodies at 4 °C overnight: Cleaved-caspase 3 (1:1000), Cleaved-caspase 9 (1:1000), Bax (1:500), NFκBp65 (1:1000; AbCam), GAPDH (1:1000), Lamin B1 (1:500) (all from Cell Signaling Technology, Inc, Danvers, MA, USA, Cell Signaling Technology), p21 (1:1000; Merck Millipore), and p53 (1:2000; Abcam, Cambridge, UK). Then membranes were incubated with horseradish peroxidise-conjugated goat anti-mouse or anti-rabbit IgG secondary antibodies (1:2000; Santa Cruz Biotechnology, Inc., Dallas, Texas, USA) at room temperature for 1 h. Protein bands were visualized using an enhanced chemiluminescence system (Luminata™ Forte, Western HRP substrate; Millipore), acquired with the ChemiDoc™ MP System (Bio-Rad Laboratories) and quantified with the ImageJ software.

### 4.8. Real-Time PCR Analysis

In order to evaluate the effect of moringin on the expression of genes encoding for apoptosis regulatory proteins, total RNA from 24 h treated or untreated SH-SY5Y cells was extracted using TRIzol reagent, according to the manufacturer’s protocol. Then, equal amounts of total RNA (2 µg) were reverse transcribed using the High-Capacity cDNA Archive Kit (Applied Biosystems, Foster City, CA). MicroRNA levels of Bax, Bcl2, p53, p21, and Caspase 3 and 9 were analyzed by SYBR green Real-Time PCR. Quantitative PCR reactions were set up in a 96-well plate and were performed in 20 µL reactions containing 1x SYBR^®^ Premix DimerEraser™ (TaKaRa Bio Inc., Japan), 0.1 µM specific primers, and 25 ng RNA converted into cDNA. Real-Time PCR was carried out on a 7300 Real-Time PCR System with the following profile: one cycle at 95 °C for 10 min, followed by 40 cycles at 95 °C for 15 s and 60 °C for 1 min. A standard dissociation stage was added to assess primer specificity. β-Actin was used as housekeeping control. The primer sequences used for Real-Time PCR are listed in Table 1. Data were collected and analyzed using the 2^−ΔΔCT^ relative quantification method [42]. Values are presented as fold change relative to untreated cells.

### 4.9. Statistical Analysis

Data were expressed as mean ± SEM and statistically evaluated for differences using one-way analysis of variance (ANOVA), followed by the Turkey–Kramer multiple comparison test (GraphPad Prism Software for Science, San Diego, CA, USA). P-values less than or equal to 0.05 were considered significant.

## Figures and Tables

**Table 1:** Oligonucleotide primers used for Real-Time PCR.

Gene Product	Primer Sequence
p21	Forward: 5′-TTCTCCACCTAGACTGTAA-3′ Reverse: 5′-GCACCTGCTGTATATTCA-3′
p53	Forward: 5′-GTGTGGAGTATTTGGATGAC-3′ Reverse: 5′-ATGTAGTTGTAGTGGATGGT-3′
Bax	Forward: 5′-GGACGAACTGGACAGTAACATGG-3′ Reverse: 5′-GCAAAGTAGAAAAGGGCGACAAC-3′
Bcl-2	Forward: 5′-ATCGCCCTGTGGATGACTGAG-3′ Reverse: 5′-CAGCCAGGAGAAATCAAACAGAGG-3′
Caspase 3	Forward: 5′- AGCACCTGGTTATTATTCTTGG-3′ Reverse: 5′- GCTTGTCGGCATACTGTT-3′
Caspase 9	Forward: 5′- GCTCAGACCAGAGATTCG-3′ Reverse: 5′- ATCCTCCAGAACCAATGTC-3′
β-Actin	Forward: 5′-TTGTTACAGGAAGTCCCTTGCC-3′ Reverse: 5′-ATGCTATCACCTCCCCTGTGTG-3′

## Abbreviations

GLs	glucosinolates
ITCs	isothiocyanates
MTT	3-(4,5-dimethylthiazol-2-yl)-2,5-diphenyltetrazolium bromide
LDH	lactate dehydrogenase
NF-κB	nuclear factor kappa-light-chain-enhancer of activated B cells
GMG	glucomoringin
HPLC	high-performance liquid chromatography
Myr	myrosinase
PI	iodide propidium
PBS	phosphate buffer saline
